# Transplant of stunned donor hearts rescued by pharmacological stress echocardiography: a “proof of concept” report

**DOI:** 10.1186/1476-7120-11-27

**Published:** 2013-08-02

**Authors:** Tonino Bombardini, Sonia Gherardi, Ornella Leone, Rosa Sicari, Eugenio Picano

**Affiliations:** 1 CNR, Institute of Clinical Physiology, Pisa, Italy; 2 Cardiology Division, M. Bufalini Hospital, Cesena, Italy; 3 Department of Pathology, University of Bologna, Bologna, Italy

**Keywords:** Heart transplant, Heart donor shortage, Stress echocardiography, Reversible wall motion abnormalities, Early graft failure

## Abstract

**Background:**

Due to the shortage of donor hearts, the criteria for acceptance have been considerably expanded. Hearts with regional or global left ventricular dysfunction are excluded from donation, but stress echo might be useful to identify patients with reversible wall motion abnormalities, potentially eligible for donation.

**Methods:**

Six marginal candidate donors (mean age, 40 ± 13 years; three men) were enrolled. Resting echocardiography showed in all subjects a LV ejection fraction ≥ 45% (mean 51 ± 5%), but multiple risk factors were present. All donors had either global or discrete wall motion abnormalities: Wall Motion Score Index (WMSI) rest = 1.33 ± 0.25. Stress echocardiography was performed with the dipyridamole high dose of 0.84 mg/kg given over 6 min.

**Results:**

The stress echo results were abnormal in three donors (WMSI rest = 1.51 ± 0.19 vs peak = 1.41 ± 0.30). These hearts were excluded from donation and cardiac pathology verification was available in two cases of confirmed LV myocardial fibrosis and/or severe coronary stenosis. The remaining three hearts improved during stress (WMSI rest = 1.15 ± 0.13 vs peak = 1.04 ± 0.06) and were transplanted uneventfully. Recipients (three males, mean age 53 ± 4 years) underwent post-TX coronary angiography, IVUS and endomyocardial biopsies. No recipient had primary graft failure, and all showed normal coronary angiography and normal LV function (EF = 57 ± 6%; WMSI = 1 ± 0) at 1-month post-TX. The recipients were alive at 12-month median follow-up.

**Conclusions:**

Dipyridamole stress echo performed in brain-dead potential donors with LV resting global or discrete wall motion abnormalities identifies hearts with severe morphologic abnormalities excluded from donation (with fixed response during stress echo) from hearts eligible for donation, showing improvement in regional wall motion during stress (viability response) and normal function and coronary anatomy following transplantation.

## Background

Brain death is a hostile environment for the donor heart and undoubtedly contributes to the occurrence of primary graft failure after HT. Donor heart dysfunction results from the “catecholamine storm” (hypertension, tachycardia, and intense vasoconstriction) that produces an increase in myocardial oxygen demand and potentially myocardial ischemia. These phenomena may mediate myofibrillar degeneration, a process characterized by injury and death of myofibers in a hypercontracted state [1]. Myocardial injury interacts with other factors, increasing the probability of post-operative primary graft dysfunction [2]. Donor risk factors known to be associated with early graft failure include higher doses of inotropic support and longer ischemic time, depressed systolic function (especially discreet wall motion abnormalities), and older donor age [3].

As far as function is concerned, a donor heart should not be used in the presence of intractable ventricular arrhythmias, the need for excessive inotropic support, discreet wall motion abnormalities on echocardiography or LVEF < 45% despite optimization of hemodynamics with inotropic support [2]. Given that a single echocardiographic assessment may be inaccurate or may fail to predict long-term ventricular contractile function, failure to use a donor heart because of the initial ejection fraction alone is not justified. Hemodynamic and metabolic management should be performed before the organ is declined, when donor left ventricular dysfunction is present [4,5]. Recent studies have shown that pharmacological stress echo is feasible for recruiting older donor hearts that were excluded simply because of the age limit in patients with normal resting function [6,7]. Pharmacological stress echo might be a feasible technique in intensive care units, to distinguish between hearts with reversible (eligible for transplant) vs non-reversible left ventricular segmental wall motion abnormalities. A practical advantage is the 2011 Italian National Institute of Health approval of echo stress methodology for heart donor selection in Italy with a second-opinion telemedicine system from the core echo lab, IFC, Pisa [8].

Aim of this study was to evaluate the usefulness of dipyridamole stress echocardiography in selecting for heart TX hearts with “standard” donor age but with multiple risk factors for early graft failure and discreet LV wall motion abnormalities in resting echocardiogram. The study hypothesis was that hearts with fixed abnormalities were not eligible as proven at cardio-autoptic verification, whereas those with reversible abnormalities were potentially eligible for transplantation as shown by subsequent angiographic evaluation in transplanted hearts.

## Methods

### Marginal donor recruitment

After legal declaration of brain death, marginal donors underwent baseline echocardiography for evaluation of regional wall motion, global ventricular function, and ventricular mass, according to American Society of Echocardiography recommendations [9].

### Donor selection by stress echocardiographic criteria

*S*tress echocardiography was performed following the European Association of Echocardiography and American Society of Echocardiography [10,11] protocol, using dipyridamole (0.84 mg/kg over 6 min). Echocardiographic images were continuously recorded and intermittently digitized. Regional wall motion score index was assessed and graded on a scale from 1 (normal) to 4 (dyskinetic) in each of the 17 segments at rest and after stress. LV wall motion score index was calculated by summing the scores and dividing the sum by 17. By definition, donors with abnormal stress echocardiographic results had fixed wall motion abnormalities and/or stress-induced wall motion abnormalities. We also considered changes in LV volumes as an index of global dysfunction, and pressure and volume changes as an index of LV elastance [6,12]. At baseline and peak stress, the projections of the four chambers and of the two apical chambers were recorded to obtain LV end-systolic volume using the biplane Simpson rule [9] to calculate LV elastance (the ratio of systolic pressure by cuff sphygmomanometry to LV end-systolic volume) [12]. A decrease in LV elastance during stress was considered abnormal. In a previous study, this response was shown to be associated with moderate to severe coronary or myocardial abnormalities on cardiac autopsy verification [13]. Potential donors with normal stress echocardiography are considered suitable donors; recipients follow a routine treatment. We accepted a priori four stress echocardiographic criteria excluding a heart from eligibility as a donor: (1) new regional wall motion abnormalities (regional wall motion score > 1.0 in at least one segment), (2) abnormal regional or global LV dysfunction with fixed response to stress; (3) negative LV elastance variation during stress (stress value less than resting value), and (4) submaximal stress prematurely stopped because of non-diagnostic limiting effects (e.g., hemodynamic instability or hypotension with a decrease > 40 mmHg in systolic or diastolic blood pressure) before completion of the infusion. Each of the four criteria had a different rationale and target: a new-onset regional wall motion abnormality is a highly specific sign of a significant epicardial artery stenosis [10,11]; a fixed response of abnormal regional or global LV function is a specific sign of an irreversibly damaged heart that will not recover, due to extensive necrosis or scarring, and is not eligible for transplant; a lack of hyperkinetic response with no increase in pressure/volume index is a sensitive marker of underlying cardiomyopathy [6], and a submaximal test loses diagnostic and prognostic power and falls within a gray zone unacceptable in the transplantation setting [10,11]. Eligible organs were considered for transplantation [14]. Hearts that were not eligible underwent pathologic examination [13].

### Transplantation of eligible hearts

Eligible hearts (with normal echocardiographic findings) were retrieved using a standard technique and preserved with cold cardioplegic arrest and topical hypothermia. Primary graft failure after HT was defined as need for immediate post-HT mechanical circulatory support [3]. The recipients followed routine treatment and follow-up procedures. They underwent coronary angiography and intravascular ultrasound at the first month [3]. Focal and non-circumferential atherosclerosis with 50% stenosis in proximal segments of at least one coronary vessel was regarded as native and donor-transmitted coronary atherosclerosis [15]. Endomyocardial biopsies (EMBs) were taken for post-transplant rejection status surveillance. All transplanted hearts were followed, and any adverse event was monitored.

### Anatomic-pathologic study of non-transplanted hearts

Hearts deemed unsuitable as a result of stress echocardiographic results were removed from donors and sent to the Pathology Department Center for a very detailed macroscopic and microscopic study [13]. Autopsies were performed by an experienced cardiac pathologist. Coronary atherosclerosis was graded as absent, subcritical, or significant. In accordance with the criteria of cardiac catheterization given above, significant atherosclerosis was defined as focal and non-circumferential atherosclerosis with 50% stenosis in proximal segments of at least one coronary vessel.

### Statistical analysis

SPSS version 11 for Windows (SPSS, Inc., Chicago, IL, USA) was used for statistical analyses. The statistical analyses included descriptive statistics (frequencies and percentages for categorical variables and mean ± standard deviation for continuous variables). *P* values < .05 were considered statistically significant.

## Results

Six marginal candidate donors (mean age, 40 ± 13 years; three men) were enrolled (Table 1). The causes of death were head trauma in two, cerebral vascular accident in three, and cardiac arrest in one. Resting echocardiography showed in all a LVEF ≥ 45%, mean value = 51 ± 5%, but multiple risk factors were present: all were heavy smokers, three had elevated cardiac markers, three had central venous pressure > 12 mmHg, three had need for excessive inotropic support, all six had either global or discrete wall motion abnormalities, with WMSI at rest = 1.33 ± 0.25. Stress echocardiography was performed with high-dose dipyridamole (0.84 mg/kg over 6 min).

**Table 1:** Donor hearts rescued by stress echocardiography

**Donor**	**#1**	**#2**	**#3**	**#4**	**#5**	**#6**
Age (years)	53	32	50	20	45	36
Sex	Female	Female	Female	Male	Male	Male
**Medical history**						
Brain death cause	Intra-axial hemorrhage	Head injury	Subarachnoid hemorrhage	Cardiac arrest (37 min)	Head injury	Subarachnoid hemorrhage
Smoking	Yes	Yes	Yes	Yes	Yes	Yes
**Intensive Care Unit data**						
Troponin T (μg/L)	*0.1*	*0.01*	*0.01*	15*	152*	1073*
Central Venous Pressure (mmHg)	16*	*-*	*7*	*-*	13*	15*
Length of stay before death (days)	*5*	*1*	*1*	*-*	*2*	*3*
Noradrenaline (μg/kg/min)	0.6* Dopamine 10	0.28*	*0.02*	*0.1*	*-*	0.14* + dobutamine 2.8
**RESTING Echo**	Apical Hypokinesia*	Anterior-septal Hypokinesia*	Apical Akinesia*	Mild LVEF reduction*	Septal Hypokinesia*	Lateral inferior Hypokinesia*
LVEF, rest (%)	51*	46*	55	49*	60	47*
LV mass index (g/m^2^)	145*	89	112*	89	133*	102
WMSI, rest	1.29*	1.59*	1.65*	1	1.18*	1.26*
**Stress echo**						
Dipyridamole infusion (min)	6	6	4*	6	6	6
LVEF, peak (%)	60	56	59	60	75	55
WMSI, peak	1.12*	1.41*	1.71*	1	1	1.11*
Δ WMSI	- 0.17	- 0.18	+ 0.06*	0	- 0.18	- 0.15
ΔESP/ESVi (mmHg/mL/m^2^)	Negative* (- 0.21)	Flat* (0.08)	Negative* (-1.7)	Positive (+0.94)	Positive (+1.2)	Flat (0)
**Autopsy findings**	Mild DCM*	-	CAD, 90% LAD coronary stenosis*	-	-	-
Histology	Replacing interstitial fibrosis	-	Coagulative necrosis, colliquative myocytolysis	-	-	-
**Heart TX**	-	-	-	YES	YES	YES
Cold ischemia time (min)				180	172	150
**Recipient**						
Age/sex				56/M	48/M	55/M
Disease				Ischemic DC	Congenital HD	HCM
UNOS Status				2	2	2
Post-TX angiography				Normal	Normal	Normal
Intravascular Ultrasound-intimal thickness				-	LAD focal	0.6 mm LAD
Month 1 LVEF (%)				50	62	58
Post TX endomyocardial biopsies				regular myocardium	isolated foci of ischemic per transplant injury	regular myocardium
**Follow-up months**				26 alive	9 alive	12 alive

*Not Normal.

### Abnormal stress echo response with fixed or worsening wall motion

**Figure 1:**
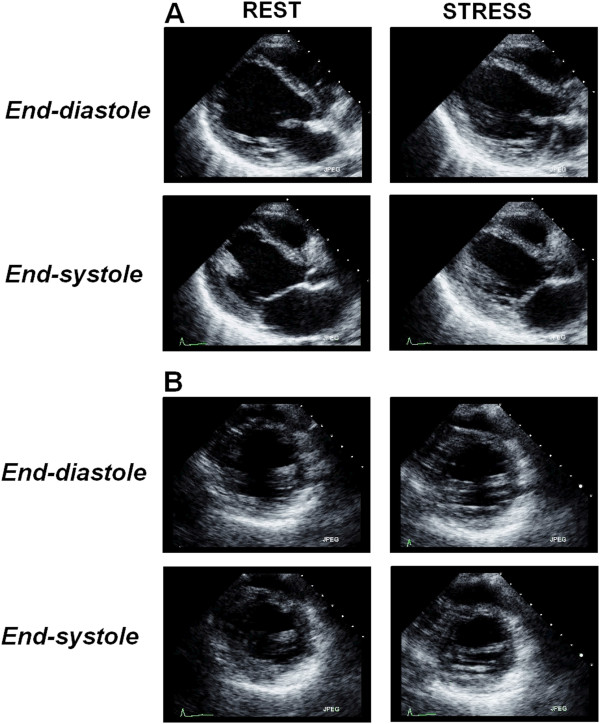
**Donor #2 in Table **1**.** An example of a stress echocardiogram in a brain-dead potential donor, showing end-diastolic and end-systolic frames at rest and following stress using Dipyridamole (0.84 mg/kg in 6’) [see Additional file 1]. Left ventricular interventricular septum and apical wall a-kinesia are shown in parasternal long-axis (panel **A**) and short-axis (panel **B**) chamber views. At peak stress an incomplete viability response is shown. The donor was considered unsuitable for heart donation.

**Figure 2:**
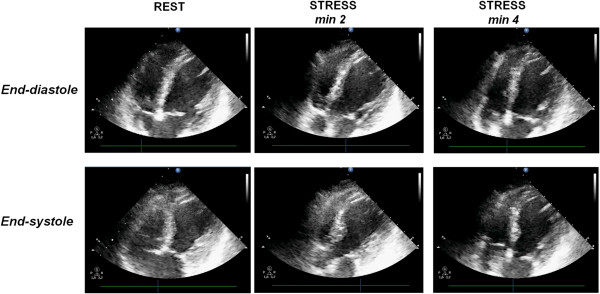
**Donor #3 in Table **1**.** An example of a stress echocardiogram with fixed abnormalities in a brain-dead potential donor, showing end-diastolic and end-systolic frames at rest and following stress using Dipyridamole. The test was prematurely stopped at 4 min due to severe systemic hypotension; WMSI rest = 1.65; WMSI = 1.71 at stop stress [see Additional file 2]. The pressure volume relation was negative with the ΔESP/ESV value = - 1.7 mmHg/mL/m^2^. The heart was sent to the pathology department for detailed macroscopic and histology examination.

**Figure 3:**
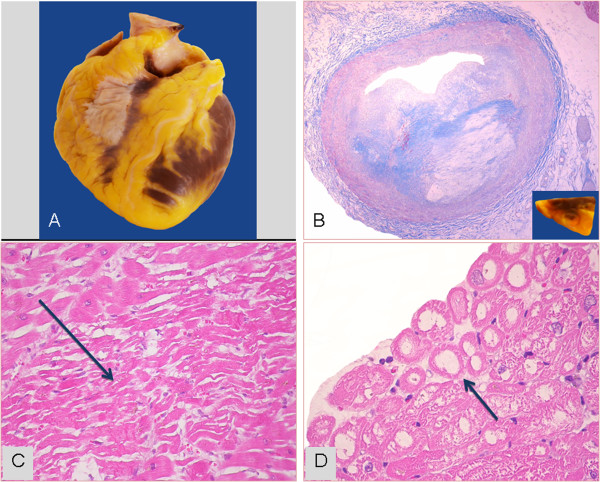
**The donor heart unsuitable for transplant of Figure **2**. A)** Macroscopic aspect of donor heart. **B)** Fibro-lipidic plaque narrowing (≈90%) the lumen of left anterior coronary artery (Mallory trichrome stain; original magnification 25x); in the inset, the corresponding macroscopic sample. **C)** Focus of myocardial coagulative necrosis (arrow) in the left ventricle (Hematoxylin-Eosin stain; original magnification 200x). **D)** Subendocardial coagulative myocytolysis (arrow) in the septal myocardium (Hematoxylin-Eosin stain; original magnification: 400x).

The stress echo results were abnormal in three donors: WMSI peak = 1.41 ± 0.30, with a flat-negative contractile reserve (Figures 1 and 2) (Additional files 1 and 2). At autopsy study (available in two), the donor with incomplete recovery of apical hypokinesia showed myocardial fibrosis with a mild DCM aspect (Table 1, donor #1); the donor with worsening of apical akinesia (Table 1, donor #3) showed a 90% LAD stenosis, multiple foci of coagulative necrosis associated with diffuse coagulative subendocardial myocytolysis (Figure 3).

### Normal stress echo response with improvement in wall motion

**Figure 4:**
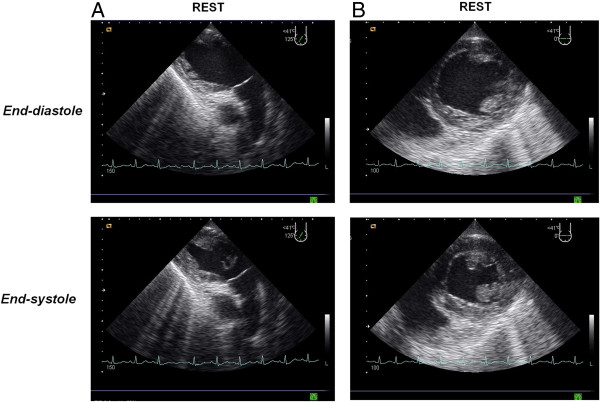
**Donor #6 of Table **1**.** Two-dimensional transesophageal echocardiogram recorded immediately after “explosive” subarachnoid hemorrhage accompanied by extreme systolic hypertensive response with systolic blood pressure ≈ 300 mmHg and acute pulmonary edema. The donor had discrete left ventricular dysfunction with hypokinesia involving the inferior and lateral LV walls [see Additional file 3].

**Figure 5:**
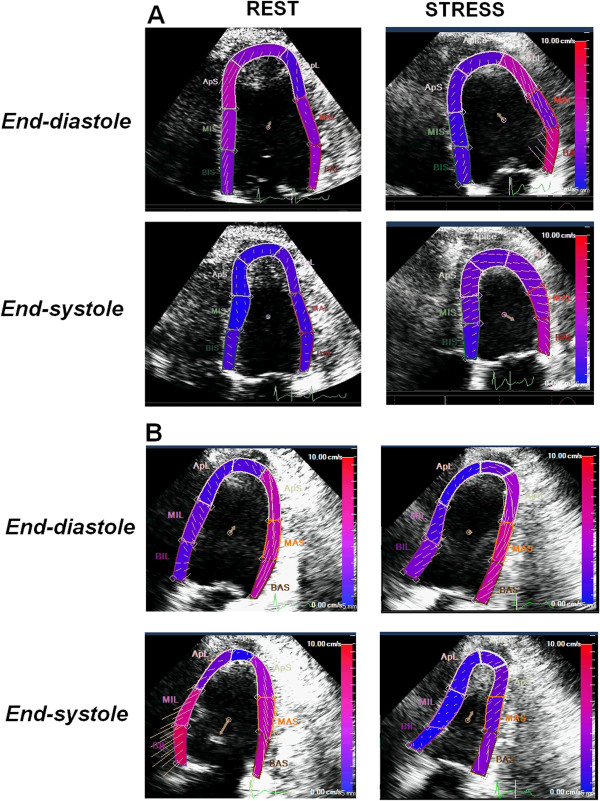
**Donor #6 of Table **1**.** The same donor as in Figure 4. Two days after the subarachnoid hemorrhage and after brain death, the donor underwent a transthoracic dipyridamole stress echo (0.84 mg/kg in 6’) [see Additional file 4]. Left ventricular lateral wall hypokinesia and inferior wall akinesia are shown in 4-chamber (panel **A**) and 3-chamber (panel **B**) views at baseline (left panels). At peak stress a viability response is shown with recovery of lateral and inferior wall motion (right panels). The donor was considered suitable for heart donation and the heart was transplanted.

**Figure 6:**
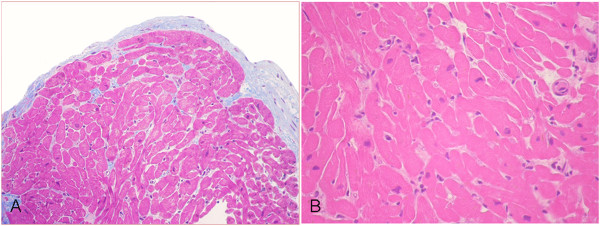
**The donor heart of donor #6 (Table **1**) after heart transplant.** In the figure, images from the first two endomyocardial biopsies (EMBs) performed on the 7^th^ (panel **A**) and 15^th^ (panel **B**) days after surgery, where regular myocardium is shown. In the EMBs taken for post-transplant rejection status surveillance, no significant ischemic peritransplant injury was noted, a finding usually seen in biopsies during the first 6 weeks after transplantation.

**Figure 7:**
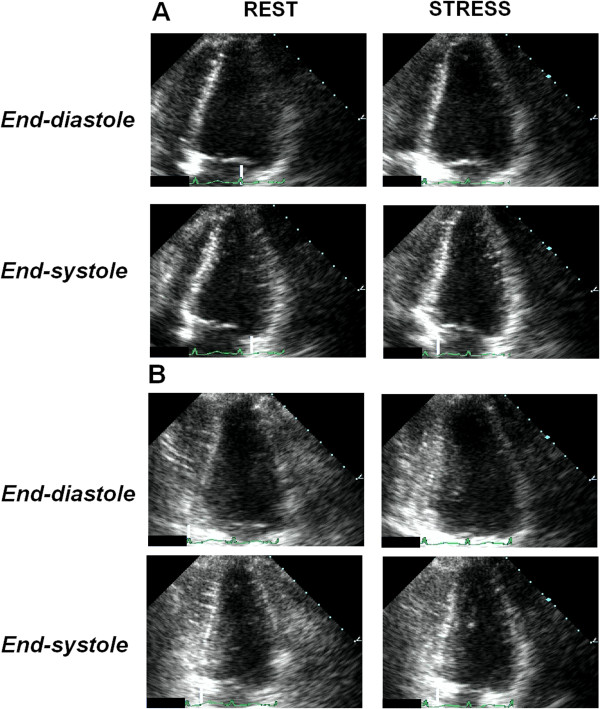
**Donor #4 of Table **1**.** Transthoracic pharmacological stress echo test using dipyridamole (0.84 mg/kg in 6’) in a potential donor with prolonged (37 min) cardiac arrest at death [see Additional file 5]. The donor had normal response with normal regional wall motion during pharmacological stress echo. (panel **A**, 4-chamber views; panel **B**, 2-chamber views). The pressure/volume relation was positive and the individual was considered a suitable donor.

**Figure 8:**
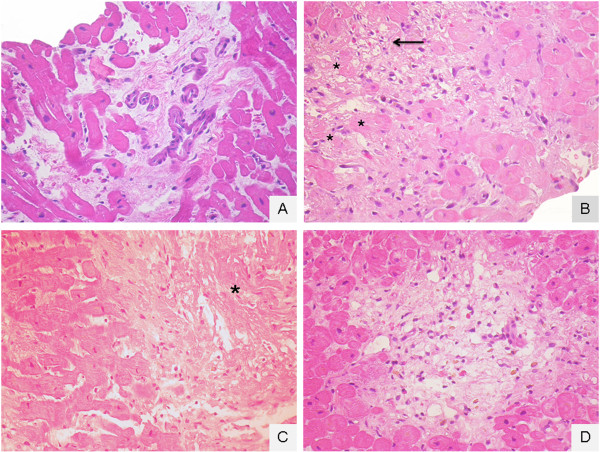
**Recipient of donor #5 in Table** 1**.** Ischemic peritransplant injury in the first 4 endomyocardial biopsies (EMBs) taken for post-transplant rejection status surveillance. **A** (12^th^ day after surgery): generic interstitial oedema; **B** (19^th^ day after surgery): a focus of peritransplant injury with hypereosinophilic cytoplasm myocites (asterisk) or with coagulative myocytolisis (arrow); **C** (26^th^ day after surgery): necrotic, evanescent (disappearing) and fragmented myocytes (asterisk); **D** (36^th^ day after surgery): a focus of resolving peritransplant injury with regional loss of myocytes, loose substitutive connective tissue/granulation tissue and pigment-laden macrophages.

The remaining three hearts showed reversible left ventricular resting abnormalities (Figures 4, 5, 6 and 7) (Additional files 3, 4 and 5). WMSI rest = 1.15 ± 0.13 vs peak = 1.04 ± 0.06. All had positive contractile reserve, with LV elastance increase during stress. These three hearts were transplanted uneventfully and underwent standard post TX coronary, angiography, IVUS and endomyocardial biopsies. The recipients were male, age 53 ± 4 years. No recipient had primary graft failure and all showed normal coronary vessel at 1-month post-TX coronary angiography: left ventricular function was normal at 1 month post-TX (LVEF = 57 ± 6%; WMSI = 1 ± 0). In the EMBs taken for post-transplant rejection status surveillance, no significant ischemic peritransplant injury was noted in two recipients (Table 1, donors #4 and #6) (Figure 6). The recipient of the donor heart with mild left ventricular hypertrophy and reversible septal hypokinesia (Table 1, donor #5) showed at EMBs mild peritransplant injury (Figure 8). The recipients were alive at 12-month median follow-up.

## Discussion

Heart transplantation is an established procedure in end-stage heart failure patients and results in satisfying long-term results. However, this surgical therapy has been limited by a severe and incremental donor organ shortage in the last few years. Therefore, adequate and optimal utilization of all suitable donor organs is mandatory for increasing graft availability. Evidence exists that certain ‘standard’ donor criteria can be significantly liberalized to increase the available donor pool by accepting ‘Marginal Donors’ who would be declined as potential organ donors under conventional transplant guidelines. If echocardiography is the initial assessment investigation, echocardiographically detected left ventricular systolic dysfunction in the absence of a history of heart disease is the single most common cause for non-transplantation of an organ [16]. However, ventricular dysfunction may be transient [17], and arbitrary thresholds of LV function may exclude hearts that could be resuscitated to transplantable status. Recent studies have shown that pharmacological stress echo (with dipyridamole or dobutamine) is a feasible tool for recruiting older donor hearts for transplant, excluded on the basis of age. The ability of pharmacologic stress testing to select these aged donors has been documented by the pathological findings in refused hearts [13] and by the clinical, angiographic and prognostic data in successfully transplanted hearts [6]. The exclusion criterion based on the presence of wall motion abnormalities is potentially overcome by the application of stress echocardiography that may transiently reverse regional dysfunction and identify the subset with a likelihood to recover over time. Such an approach has been widely demonstrated in large multicenter studies showing that the transient improvement induced by either a vasodilator or inotropic response was translated into a stable recovery and a better outcome in stunned or hibernated hearts [11,18,19]. In a large subset of brain-dead donor hearts, left ventricular performance is reduced because the myocardium is regionally stunned or hibernating rather than irreversibly infarcted or fibrotic. The detection of reversible dysfunctional myocardium is clinically relevant, as regional or global left ventricular function will improve after transplant. The phenomenon of troponin elevation and cardiac dysfunction in the donor may be transient, suggesting altered sarcolemmal integrity rather than myocyte necrosis [5].

With pharmacological stress, the principle (i.e., the underlying physiological marker) of the test relies on the demonstration of residual contractile reserve in a basally dysfunctional region; improved myocardial thickening of segments that are dyssynergic in resting conditions is a sign of viability, whereas necrotic segments show no functional improvement. Such reserve can be elicited through a flow-mediated increase in contractile function linked to endogenous adenosine accumulation achieved by intravenous infusion of dipyridamole.

### Comparison with previous studies

Left ventricular dysfunction is a common finding in patients with intracranial pathologies and brain stem death. In 147 patients with subarachnoid hemorrhage, global or regional LV dysfunction was found in 30 (20%) patients on echocardiography. Regional wall motion abnormalities tended to cover multiple arterial territories and occurred in the absence of coronary artery disease [20]. This frequency has been confirmed in a retrospective study of 66 patients with brain stem death, 28 (42%) of whom were found to have global or segmental LV dysfunction that was not predicted by clinical and electrocardiographic examination [21]. Secondly, left ventricular dysfunction documented on echocardiography in heart donors with BSD does not appear to correspond to any demonstrable pathological abnormality at postmortem [22]. Thirdly, there is clear evidence not only that donor hearts with mild abnormalities in LV function on rest echocardiography can be successfully transplanted but also that donor hearts with more severe regional wall abnormalities may improve immediately post-transplant [23]. The stress echo approach might also be applied to these potential heart donors with left ventricular wall motion abnormalities, since viability response during stress echo effectively recognizes viable tissue with non-fixed response, as opposed to necrotic response with scar and fixed wall motion abnormalities following inotropic challenge with either dobutamine or dipyridamole [24,25].

## Conclusion

Left ventricular dysfunction is common in donor hearts and in several cases does not correspond to a detectable pathological abnormality. Abnormal regional or global resting LV dysfunction is a functionally heterogeneous entity, encompassing patients both unsuitable and suitable for donation. The former group shows fixed or worsening (abnormal) wall motion response during stress echo, and significant myocardial and/or coronary alterations at pathology verification. The second group shows normal (reversible, viability) wall motion response during stress echo, and absence of myocardial and/or coronary alterations at angiography verification, and can be transplanted uneventfully. The promising data obtained in this proof-of-concept study should now be substantiated in a larger series in order to change current guidelines ruling out donation on the basis of probably too-restrictive criteria of global or discreet regional wall motion abnormalities.

## Abbreviations

BSD: Brain stem death; DIP: Dipyridamole; EF: Ejection fraction; EMBs: Endomyocardial biopsies; HT: Heart transplant; IVUS: Intra Vascular ultra sound; LV: Left Ventricular; TX: Transplant; WMSI: Wall motion score index.

## Competing interests

The authors declare that they have no competing interests.

## Authors’ contributions

TB conceived this report, performed the data analysis, and drafted the manuscript; OL performed the anatomic and histology examinations of non transplanted and transplanted hearts and revised the manuscript; SG was responsible for data collection and revised the manuscript; EP and RS gave a contribution to the preparation of study design, data discussion, and critical revision of the manuscript. All authors read and approved the final manuscript.

## Authors’ information

TB, Scientific Coordinator of the CCM project n. 48 “Aged Donor Heart Rescue by Stress Echo – ADONHERS” Institute of Clinical Physiology, National Research Council, Pisa, Italy. SG, Cardiology Division and Intensive Care Unit, M. Bufalini Hospital, Cesena, Italy. OL, Pathology Department, University of Bologna, Italy. RS, is the Head of the Echocardiography Lab, IFC, CNR, Pisa. EP, Director IFC, CNR, Pisa, Italy.

## Supplementary Material

Additional files 1 **Quad screen DIP stress echo of a potential heart donor with discrete wall motion abnormalities at rest showing incomplete improvement in regional wall motion during stress.** Upper panels rest; lower panels peak stress. Click here for file

Additional files 2 **LV apical wall akinesia is shown at baseline (left upper panel) and 1-min stress (right upper panel).** At 3- and 4-min stress (lower panels) a worsening of LV function is shown accompanied by hemodynamic instability. Click here for file

Additional files 3 In both the two-chamber (upper panel) and short-axis (lower panel) views note the inferior and lateral left ventricular dysfunction. Click here for file

Additional files 4 **Left ventricular lateral wall hypokinesia and inferior wall akinesia are shown in 4- and 3-chamber views at baseline (left and right upper panels).** At peak stress a viability response is shown with recovery of normal lateral and inferior wall motion (lower panels). Click here for file

Additional files 5 Quad screen DIP stress echo: global and regional wall motion is shown in 4- and 2-chamber views at baseline (upper panels) and at peak stress (lower panels). Click here for file
